# Role of the Flat-Designed Surface in Improving the Cyclic Fatigue Resistance of Endodontic NiTi Rotary Instruments

**DOI:** 10.3390/ma12162523

**Published:** 2019-08-08

**Authors:** Gianluca Gambarini, Gabriele Miccoli, Marco Seracchiani, Tatyana Khrenova, Orlando Donfrancesco, Maurilio D’Angelo, Massimo Galli, Dario Di Nardo, Luca Testarelli

**Affiliations:** 1 Department of Oral and Maxillo-Facial Sciences, Sapienza University of Rome, 00161 Rome, Italy; 2 Department of Anatomy, Histology, Forensic Medicine and Orthopaedics, Sapienza University of Rome, 00185 Rome, Italy

**Keywords:** nickel–titanium (NiTi), endodontics, flat design

## Abstract

The aim of this study was to investigate the role of the flat-designed surface in improving the resistance to cyclic fatigue by comparing heat-treated F-One (Fanta Dental, Shanghai, China) nickel–titanium (NiTi) rotary instruments and similar prototypes, differing only by the absence of the flat side. The null hypothesis was that there were no differences between the two tested instruments in terms of cyclic fatigue lifespan. A total of 40 new NiTi instruments (20 F-One and 20 prototypes) were tested in the present study. The instruments were rotated with the same speed (500 rpm) and torque (2 N) using an endodontic motor (Elements Motor, Kerr, Orange, CA, USA) in the same stainless steel, artificial canal (90° angle of curvature and 5 mm radius). A Wilcoxon–Mann–Whitney test was performed to assess the differences in terms of time to fracture and the length of the fractured segment between the flat- and non-flat-sided instruments. Significance was set at *p* = 0.05. The differences in terms of time to fracture between non-flat and flat were statistically significant (*p* < 0.001). In addition, the differences in terms of fractured segment length were statistically significant (*p* = 0.034). The results of this study highlight the importance of flat-sided design in increasing the cyclic fatigue lifespan of NiTi rotary instruments.

## 1. Introduction

The development of endodontic nickel–titanium (NiTi) rotary instruments have improved root canal shaping, making the treatment more feasible, repeatable, and faster [1,2,3]. Despite the superior mechanical properties of the NiTi alloy, the risk of intracanal separation of the nickel–titanium rotary instruments has been shown to increase when compared with the traditional stainless steel files [1].

Cyclic fatigue is considered one of the most relevant causes of instrument breakage [4,5,6]. Resistance to fatigue was found to be related with cross-sectional design and other factors, including type and quality of manufacturing, dimensions, taper, and heat treatment [7,8,9,10,11]. Previously published studies have shown that mechanical motion also plays an important role in improving cyclic fatigue resistance by reducing the stress applied to the instrument [12,13,14].

More recently, a new thermally treated NiTi rotary instrument, F-One (Fanta Dental, Shanghai, China), characterized by an S-shaped, flat-sided cross-section has been developed. The manufacturer claims that the S-shape and flat design confers advantages in terms of reducing blade engagement and increasing fatigue lifespan. Moreover, it is claimed that the flat design reduces stress by sweeping the debris from the flutes to the relieved area [15,16,17]. To date, no studies evaluating these instruments or the influence of flat design on the mechanical resistance of NiTi instruments are present in the literature.

**Figure 1:**
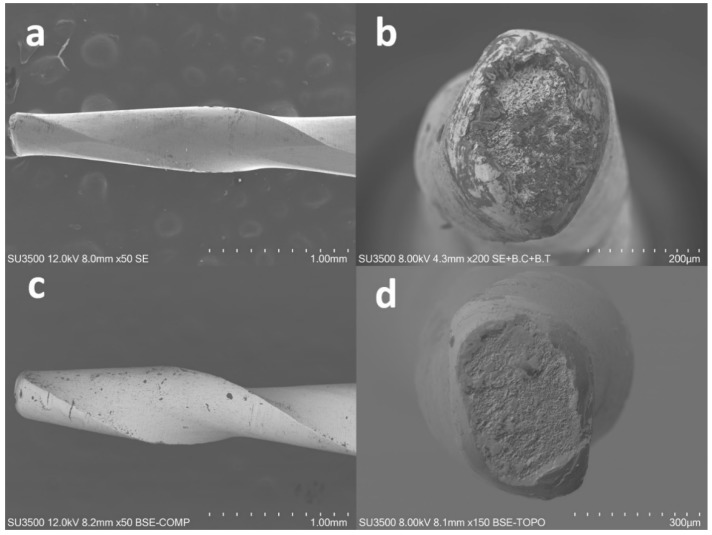
Lateral and cross-sectional aspect of flat-designed (**a**,**b**) and prototype instruments (**c**,**d**) at field emission scanning electron microscope (FE-SEM).

Since many factors contribute to cyclic fatigue resistance, to evaluate only the influence of cross-sectional design, instruments should be compared with similar ones made with the same alloy, heat treatment, and manufacturing process. In the present study, the manufacturer was asked to provide both F-One instruments and similar prototypes made with the same heat treatment and design, pitch, blade angle, and cross-section that differed only by the absence of the flat sides (Figure 1).

The aim of this study was to investigate the role of the flat-designed surface in improving the resistance to cyclic fatigue by comparing the abovementioned commercial instruments and the prototypes. The null hypothesis was that there were no differences between the two tested instruments in terms of cyclic fatigue lifespan.

## 2. Materials and Methods

A total of 40 new NiTi instruments (20 F-One and 20 prototypes) were tested in the present study. Both instruments were of the same length, 25 mm, with the same tip size #25, a taper of 4%, and made by the same heat-treated NiTi alloy—the only difference was the presence of the flat-sided design in the instruments of the test group and its absence in the control group.

All instruments had been previously inspected for macroscopic morphological defects and/or any visible signs of surface deformations using a stereomicroscope at ×20 magnification. None of them were discarded. 

All instruments were rotated with the same speed (500 rpm) and the same torque (2 N) using an endodontic motor (Elements Motor, Kerr, Orange, CA, USA).

**Figure 2:**
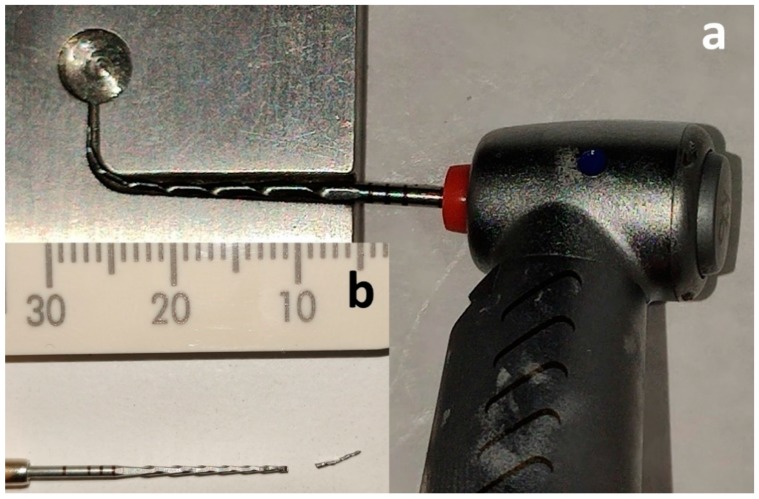
The instrument engaged in the artificial, stainless steel canal (**a**) and the fractured flat-sided instrument with its fragment (**b**).

A cyclic fatigue testing device, which had been validated in previous studies, was used [18,19]. The device consists of a mobile platform connected to an electric handpiece that allows a precise and repeatable placement of the tested instruments inside the artificial root canal and a stainless steel block containing the artificial canals (Figure 2).

The test was performed by inserting each instrument in the same stainless steel, artificial canal with a 90° angle of curvature and a 5 mm radius of curvature for the same length (18 mm). Each test was performed at room temperature (20 °C) by the same operator [20].

All instruments were rotated in a continuous rotation motion until fracture occurred. A lubricant (Super Oil, Singer LTD, Elizabethport, NJ, USA) was used to further reduce the friction between the file and the canal walls [11]. For each instrument, the time was stopped as soon as the fracture was visible and registered with a 1/100 s chronometer. The time to fracture for each instrument was recorded (TtF). Fragments were collected and measured by using a digital caliber. After the test, the instruments were inspected by FE-SEM analysis to define patterns of fracture.

Data were collected, and the mean and standard deviation were calculated. A Wilcoxon–Mann–Whitney test was performed to assess differences in terms of time to fracture (in seconds) and the length of the fractured segment (mm) between flat-designed and prototype instruments. Significance was set at *p* = 0.05. Analyses were performed with SPSS 13.0 (Incorporated, Chicago, IL, USA) for Windows.

## 3. Results

All 40 records entered the statistical analysis. The differences in terms of seconds to fracture between instruments were statistically significant (*p* < 0.001) with a mean of 64 ± 15.63 s for the flat-designed ones and 38.4 ± 11.82 s for the prototypes. In addition, the differences in the length of the fractured segment (FL) between the flat-designed (5.9 ± 1.3 mm) and the prototypes (5.3 ± 0.44 mm) were statistically significant (*p* = 0.034) (Table 1).

**Figure 3:**
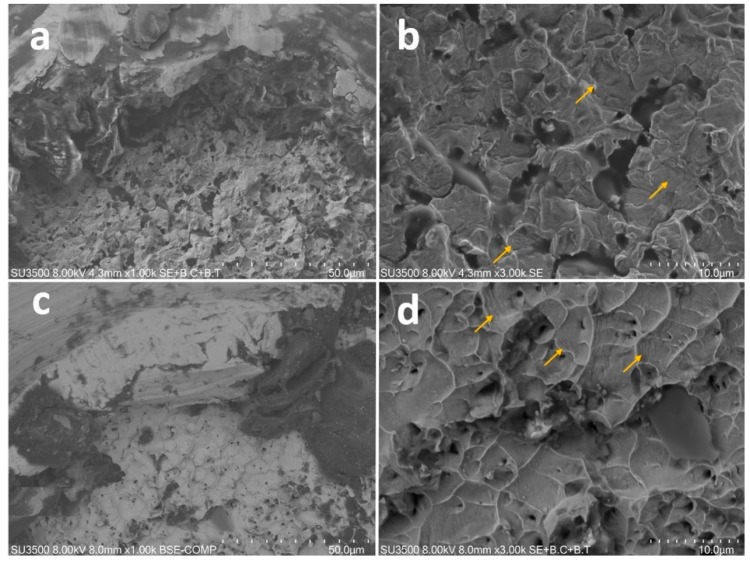
Microscopical aspect of the flat-designed (**a**,**b**) and prototype (**c**,**d**) fractured surface at different FE-SEM magnifications. Yellow arrows indicate fatigue striations that could be appreciated at 3000× (**b**,**d**).

SEM fractographic analysis showed signs of ductile fracture in all specimens—fatigue striations were clearly visible at higher magnifications (Figure 3).

## 4. Discussion

The instruments compared in the present study were characterized by the same tip size #25, the same 4% constant taper, and the same cross-sectional, S-shaped design—the only difference was the presence of the flat-sided design of the F-One file. Both instruments were rotated with the same motor, motion, speed, and torque to eliminate possible kinematic influences [12]. To mimic performance in severe curvature, a challenging 90° curvature was chosen for the tests. High degree curvatures have been shown to be one of the most common causes of clinical fracture [21,22].

**Figure 4:**
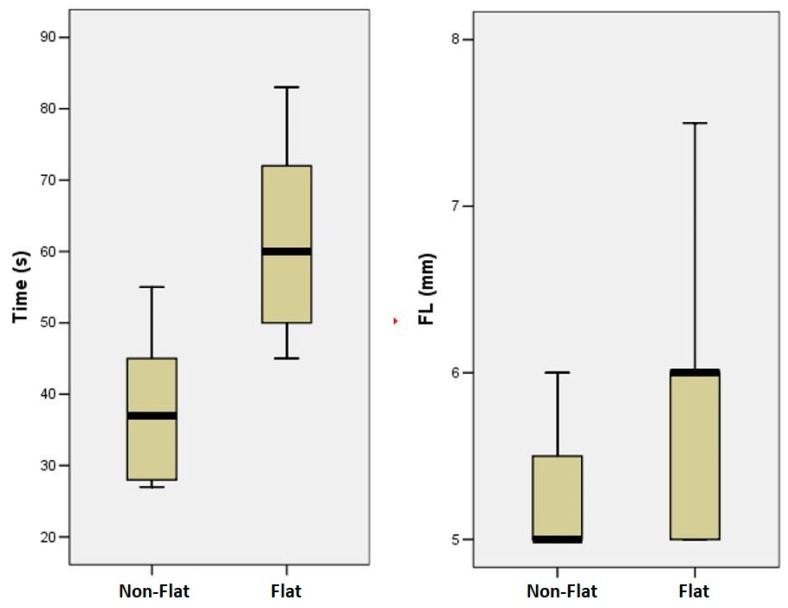
Differences in terms of time to fracture and length of the fractured segment between instruments. Box plots representing the differences between the two groups for each aspect.

The results showed significant differences between the two tested instruments—the F-One flat-designed instruments showed a higher resistance to cyclic fatigue (Figure 4). The null hypothesis was rejected, because the results of this study highlighted the importance of flat-sided design in increasing cyclic fatigue lifespan of NiTi rotary instruments. Two factors can explain this result—the reduced metal mass and the consequently increased flexibility. Data of the present study are in accordance with studies demonstrating that the resistance of rotary instruments is influenced by the quantity of metal mass—an increased mass resulted in a higher torsional resistance but a smaller resistance to flexural stresses [23,24].

The S-shaped, cross-sectional design is characterized by a smaller cross-sectional metal mass compared to other designs; in the present study, the mass was reduced even more by the flat-sided design. The more complex the canals are, the more relevant the influence of the mass—a more severe curvature results in a higher flexural stress [21]. Several studies have shown that the more flexible the file is, the more resistant it is to cyclic fatigue. Reduced mass was also shown to be a relevant factor in increasing the flexibility of the instruments [24,25].

In clinical practice, a flat design could also be beneficial in reducing torsional stresses, because the contact surface is smaller and less friction is produced when progressing into the canal. A reduced blade engagement and friction can also influence the cyclic fatigue study, because the instrument is less engaged in the artificial canal while rotating [5]. Several studies have shown that, if the instruments are able to move more freely or straighten during the cyclic fatigue test, the resistance can be slightly improved [26,27,28]. 

Thermal treatment has a relevant impact on the cyclic fatigue test; usually, a higher percentage of martensitic structure in the instrument may significantly influence the cyclic fatigue lifespan [29]. The same could happen when the test is performed at different temperatures [30,31]. In addition, the manufacturing process and machining may influence the instrument’s fatigue lifespan by inducing external or internal defects. Defects deriving from the manufacturing process could be responsible for the formation of microcracks on the surface—their deepening during usage will lead to the complete separation of the instrument as a result of each loading cycle [10,32,33,34].

In the present study, to avoid all these influencing factors, all tested instruments (commercial and prototype ones) were made by the same manufacturer, the same grinding machine, the same lot of NiTi wire, and the same heat treatment to properly investigate the relevance of the flat design without confounding factors. The results also showed statistical relevant differences in the fragment lengths between the two types of instruments. The flat-surfaced instruments failed at ~1 mm coronal instead of non-flat-surfaced ones, in a thicker and consequently less flexible site respect to the prototype [35].

All the specimens showed ductile fracture due to the removal of all the possible external forces, such as dentine hardness or anatomical differences, which could provide taper-lock-like phenomena and, consequently, a torsional failure [5]. The device and the methodology adopted in this study have been previously validated and special care was taken to avoid those factors which have been shown to influence the reliability of these tests, such as instrument fit, instrument insertion, and customized canals [7,9,11,19]. The results obtained in this study are due to a standardization of the procedures and the anatomical aspects of the artificial canal; they are not reproducible clinically, where the differences between the endodontic characteristics could vary considerably [36,37]. 

## 5. Conclusions

The reduction of the mass in the production of endodontic instruments revealed an improved resistance to flexural fatigue due to the major flexibility reached, when compared with similar specimens characterized by a higher volume of alloy. The flat-designed instruments failed at a more coronal position in respect to the prototypes due to the major mass of alloy at this level. Studies are needed to better evaluate the clinical advantage of this type of cross-sectional design on torsional resistance, operative torque, cutting efficiency, and debris removal.

## Figures and Tables

**Table 1:** Recorded data of the instruments inserted in an 18 mm artificial canal (90° angle and 5 mm radius) at the speed of 500 rpm. Time to fracture (TtF) and length of the fractured segment (FL), with means and standard deviations.

Specimen	25.04 F-One		25.04 Prototype
	TtF (s)	FL (mm)	FL (mm)
1	45	5	5
2	50	5	5
3	60	7.5	6
4	72	6	5.5
5	83	6	5
6	45	5	5
7	50	5	5
8	60	7.5	6
9	72	6	5.5
10	83	6	5
11	45	5	5
12	50	5	5
13	60	7.5	6
14	72	6	5.5
15	83	6	5
16	45	5	5
17	50	5	5
18	60	7.5	6
19	72	6	5.5
20	83	6	5
			
MEAN	62	5.9	5.3
SD	15.63649577	1.302469508	0.45
